# Integrative social cognition remediation with social skills training for adults: PICSIS – pilot study in autism and schizophrenia

**DOI:** 10.3389/fpsyt.2025.1688937

**Published:** 2026-01-12

**Authors:** Maxime Visser, Amandine Béasse, Hala El Gholabzouri, Marie Chenault, Josselin Didou, Milena Kostova, Yannick Morvan, Mauricette Mendy, Isabelle Amado, Mona Moualla

**Affiliations:** 1 Centre Ressource île de France, de Remédiation cognitive, Réhabilitation Psychosociale et Rétablissement (C3RP), Paris, France; 2 Groupe Hospitalier Universitaire (GHU) Paris Psychiatrie Neurosciences, Paris, France; 3 Laboratoire Paragraphe (UR 349), Université Paris 8, Saint-Denis, France; 4 Laboratoire Neuropsychopathologie Cognitive : Evaluation et Traitement (NCET), Université Laval, Québec, QC, Canada; 5 Laboratoire CLInique PSYchanalyse Développement (CLIPSYD), Université Paris Nanterre, Nanterre, France

**Keywords:** social cognitive remediation, social cognition, ASD, schizophrenia, psychosocial therapies, cognitive remediation

## Abstract

Autism spectrum disorder (ASD) and schizophrenia share neurodevelopmental alterations, particularly deficits in social cognition (SC) that strongly influence social and functional outcomes. Although social cognition remediation (SCR) and social skills training (SST) have each shown efficacy, no validated intervention yet integrates both approaches. This open-label, prospective study examined the feasibility and preliminary effects of PICSIS (Programme Intégratif de remédiation de la Cognition Sociale et d’Interaction Sociale), a group-based program alternating 19 SCR and 11 SST sessions over 30 biweekly sessions. Eighteen clinically stable adults with ASD or schizophrenia (mean age = 35.8 ± 9.3 years) completed the intervention. Feasibility was assessed through attendance and dropout rates; clinical, cognitive, and functional outcomes were evaluated pre- and post-intervention using validated French instruments (ClaCoS battery, ERF-CS, GAF, BPRS). Attendance was high (89.4%), confirming feasibility. While clinical symptoms and global functioning did not significantly change, participants showed significant improvements in theory of mind (MASC, p = 0.033, r = 0.61), reduced hostility attribution bias (AIHQ, p = 0.009, r = 0.82), and fewer self-reported SC complaints (ACSo, p = 0.0037, r = 0.89). Functional outcomes exhibited positive, non-significant trends. These preliminary findings indicate that PICSIS is feasible, well tolerated, and associated with specific social cognitive benefits across diagnostic categories. Further randomized controlled studies with larger samples are warranted to confirm its efficacy and long-term impact.

## Introduction

Autism spectrum disorders (ASD) and schizophrenia affect approximately one in 127 adults (1), respectively, in the general population and exhibit partially overlapping clinical profiles. Both conditions are neurodevelopmental disorders characterized by cognitive impairments, including deficits in neurocognitive functioning. A core area of convergence involves social cognition, which refers to the processes underlying the perception, interpretation, and appropriate behavioral response to social information. Impairments in social cognition are consistently associated with poorer functional outcomes across both diagnostic groups (2).

A systematic review and meta-analysis directly comparing social cognition in individuals with schizophrenia and those with ASD reported broadly similar levels of impairment across the two groups (3). No significant group differences emerged in theory of mind, emotion regulation or emotion recognition, and social perception (3). Bridging the gap between autism and schizophrenia, Gur et al. (4) and, more recently, Barlati et al. (5) proposed social cognition as a key construct within the Research Domain Criteria (RDoC) framework. According to these authors, the similarity of impairments suggests that interventions proven effective in enhancing social cognition in schizophrenia may also benefit individuals with autism.

Social cognition deficits have been identified as stronger predictors of community functioning than neurocognitive impairments in schizophrenia (6–8). Although limited research has examined community-level predictors of outcomes in autistic adults, social cognition appears to play a crucial role in determining social functioning in this population as well (9). It is therefore essential to provide targeted interventions to improve social cognitive performance and, in turn, social functioning. Such personalized approaches may also enhance the characterization of individual profiles in both autism and schizophrenia (5).

The reference treatment for SCI (Social Cognitive Impairment) is social cognition remediation (SCR). In their meta-analysis, Roelofs and colleagues (10) highlighted the strengths and limitations of different SCR programs by gathering them into 3 types of programs: targeted programs, comprehensive programs and broad-based programs. Targeted programs focus specifically on a social cognition skill. An illustration could be two French programmes: ToM Remed which targets Theory of mind (ToM) deficits (11; 2010) and Gaïa (12; 2012), a program focused on Facial emotion impairment (FEI). If these methods can improve targeted skills, they may be limited in their generalization and functional impact. Moreover, training materials in these two methods are the same as those used for the assessments. Comprehensive programs can focus on several social cognition skills, such as the SCIT-S (Social Cognition Interaction Training for Schizophrenia – 13, 14) or the individual RC2S (Remédiation Cognitive de la Cognition sociale - 15; 2016) methods. Both show clear benefits on FEI, ToM and Attributional Bias (AB) (16–18), but not for Social Perception (SP), with a significant but moderate effect on social functioning. Finally, broad-based programs combine SCR with other types of psychosocial treatments such as neurocognitive remediation (NR), showing improvements in SCI except SP and are associated with greater functional improvements (10, 19). For example, the CET (Cognitive Enhancement Therapy, 20, 21) which combines SCR and NR, has demonstrated improvement on neurocognition, social cognition and social adjustment with patient with schizophrenia even 2 years after program (22, 23). An adapted version of CET for ASD also shows improvement in neurocognition but with no difference in efficacy on social cognition compared to an enriched support therapy after 9 months (21).

Social Skills Training (SST) is another type of psychosocial therapy that has shown efficacy in improving social functioning in schizophrenia (24) and autism (25). The ISST (Integrated Social Cognitive and Behavioural Skill Therapy, 26) is a broad-based program combining SCR (mostly focus on FEI) with SST with individual and group sessions. ISST provides a significant improvement in FEI performance but not on ToM performance with moderate to large effects on social functional improvements in schizophrenia (27). Meta-analytic evidence shows that SST in adults with schizophrenia provides better social outcomes and small improvements in negative and positive symptoms (28). In autism, two studies based on the PEERS program (Program for the Education and Enrichment of Relational Skills) have demonstrated enhanced social knowledge and social skills with adults (29, 30). In addition, two programs specifically targeting social skills for employment incorporating emotion regulation and role-play exercices, reported improvements in social cognition, an increase in the number of hours worked, and facilitated access to employment (31). Another study focusing on job interview skill acquisition reported improved socio-pragmatic abilities (32).

SST and SCR are therefore effective in both schizophrenia and ASD (18, 33). To our knowledge, no broad-based program mixing a comprehensive structure for social cognition combined with SST with group sessions has been yet validated. Based on this literature we built a new method which combines SST and SCR in group sessions with a mixed aim, integrating people with schizophrenia and ASD, PICSIS (Programme Intégratif de remediation de la Cognition Sociale et d’Interaction Sociale/Social cognition and social interaction integrative program).

The goal of this study was to examine the feasibility, and the primary results of PICSIS, a cognitive remediation method for social cognition built in our Resource Center for cognitive remediation, psychosocial rehabilitation and recovery (C3RP). We hypothesized that participants with schizophrenia spectrum disorders or ASD would demonstrate improvement in social cognition performance and less functional difficulties after PICSIS.

## Methodology

### Study design

The present open-label, non-randomized, prospective clinical trial was conducted at the C3RP unit attached to the GHU psychiatry and neuroscience (Sainte-Anne Hospital, Paris), between March 2023 and January 2025.

Only outpatients were recruited. Individuals were referred to the unit at the end of their hospitalization, but their first appointment took place after discharge, once they have reached a stable clinical condition with no changes in medication dosage for at least one month. Individuals with schizophrenia were referred to this unit for psychosocial therapies and rehabilitation programs (e.g., return to studies, vocational training). For autism, individuals could be referred to the C3RP: for diagnosis, using a structured interview concerning childhood and the Autism Diagnostic Observation Schedule, Second Edition (ADOS-2) (34), and to develop a personalized plan for social inclusion. Each participant underwent a medical interview, a neuropsychological evaluation, and functional assessments (JD).

### Participants

All the participants were addressed toward the C3RP unit by psychiatrists with a demand to build a social or professional inclusion project based on psychosocial therapies. We included participants who fulfilled the diagnosis of ASD or schizophrenia (DSM-5), without intellectual disability in the anamnesis, aged between 18 and 56 years old, clinically stable, fluent in French. Autism and schizophrenia diagnoses and their comorbidities (ADHD, bipolar disorder, anxiety, depression) were verified through specialized, semi-structured clinical interviews that were indexed to DSM-5 criteria. Exclusion criteria were: previous diagnosis of intellectual deficiency; taking anxiolytics or sedatives during the day (evening intake for hypnotic purposes was allowed); abuse or addiction to toxic substances; clinical instability at the time of inclusion; a modification of the drug treatment in the month before inclusion when persons were receiving medication; a lack of mastery of the French language; no participation in any social cognition remediation program during the 18 months preceding the start of PICSIS.

The rehabilitation process was initiated with a clinical, neuropsychological and functional multidisciplinary evaluation which allowed to determine if cognitive remediation for social cognition was recommended. As a specific criterium to enter in PICSIS program, individuals showed ToM impairments, objectified by at least one score below the 10th percentile on a ToM test, the Movie Assessment of Social Cognition (35, translated by 36). According to cognitive assessment, when neurocognitive impairment could impact the participation of PICSIS because of attention, organization or working memory impairments, the participant first took part in a neurocognitive remediation program, such as NEAR (Neuropsychological Educational Approach for Cognitive Remediation - Medalia and Freilich (37). Written informed consent was obtained from all participants after clear and fully explanations for the study and for the content of the PICSIS program. This research was approved by local Ethics Comity (cpp-ouest2- chu-angers, n° 2019-A00930-57) and followed the recommendations of the latest Declaration of Helsinki.

### Intervention program: the PICSIS program

**Figure 1:**
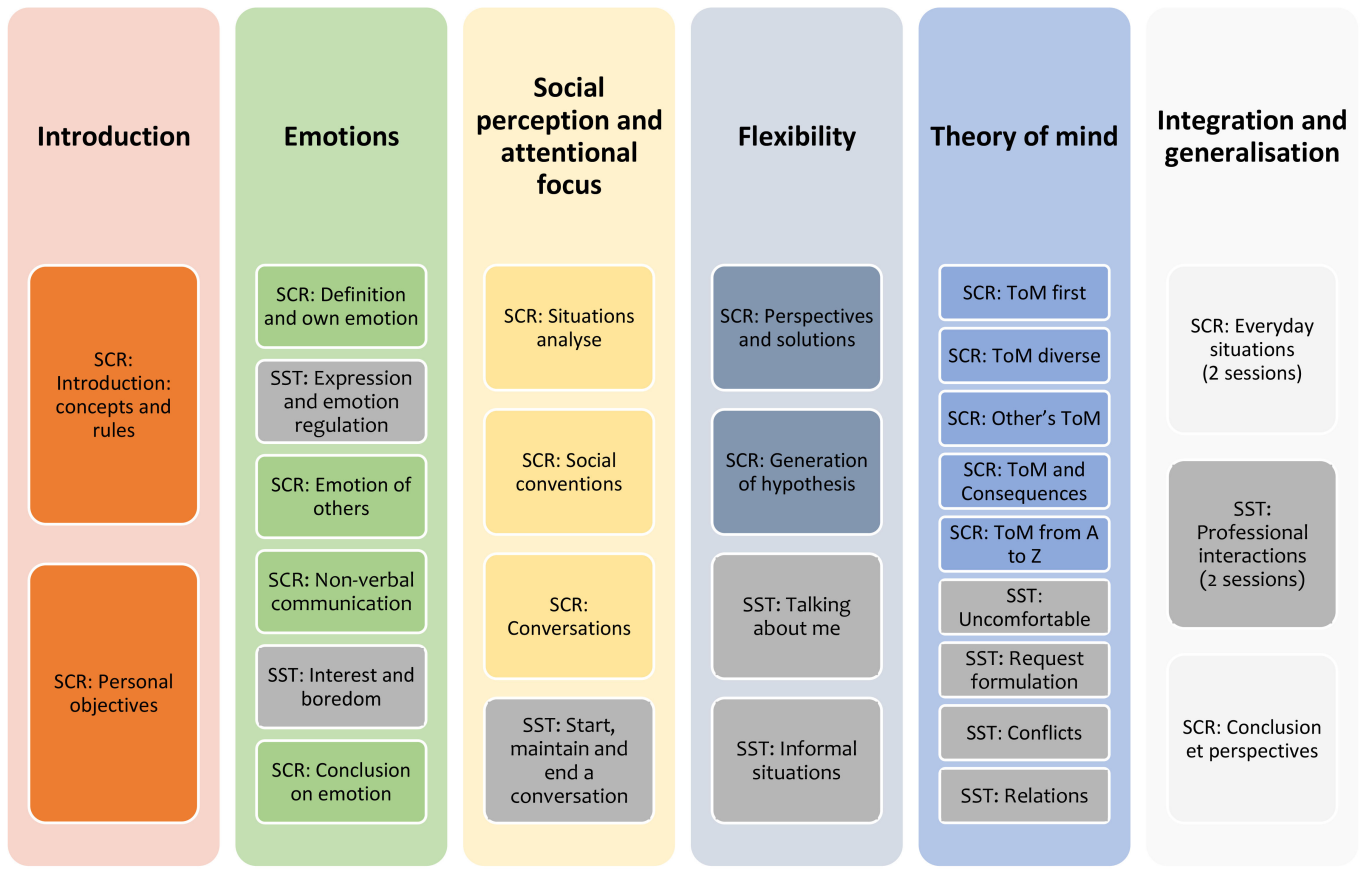
PICSIS method schedule. The shaded boxes represent the Social Skills Training (SST) sessions, and the colored boxes represent the Social Cognition Remediation (SCR) sessions.

The PICSIS program is a 30-session program alternating between 19 SCR and 11 SST sessions spread over 6 modules. The program is run in a group setting, with 2-hour-biweekly sessions co-hosted by 2 therapists. The intervention can accommodate between four and eight participants. The structure of the program is presented in **Figure 1**.

The first introductory module is designed to help each participant establish personal objectives within the domain of social cognition. The second module focuses on emotions—specifically their definition, subjective experience, facial emotion recognition, and nonverbal communication—and includes four remediation sessions and two social skills sessions. The third module targets social perception and attentional focus, covering situational analysis, social conventions, and conversational skills; it comprises three remediation sessions and one social skills session. The fourth module addresses cognitive flexibility, aiming to develop the ability to generate alternative hypotheses in social situations, and includes two cognitive remediation sessions and two social skills sessions. The fifth module focuses on theory of mind and consists of five remediation sessions and four social skills training sessions. The final module is dedicated to the generalization of acquired skills to daily life, with two social skills sessions focus on everyday situations and two sessions focused on the professional environment.

Each SCR module begins with sessions in which therapists present materials such as images, video clips, or situational vignettes. Strategies are introduced using these neutral stimuli, after which participants are systematically encouraged to apply the strategies to personal situations shared within the group. SST sessions start with a theoretical overview and the presentation of a specific methodology, followed by role-play exercises based directly on situations reported by participants. Support materials are provided, including summaries of session content and tools to guide both in-session exercises and homework assignments. This overall methodology aligns with the recommendations of Bowie et al. (38), emphasizing early and repeated problem-solving exercises grounded in real-life situations reported by users.

### Study procedures

All participants underwent clinical, social cognition and functional assessments at baseline approximately one month before the beginning of the program (T0), and one to two months after program completion (T1). Variability in these time intervals was due to the scheduling constraints of both participants and examiners. Two separate assessments were required at each time point: clinical and psychosocial functioning evaluations were conducted by psychiatrists, whereas cognitive assessments were administered by a psychologist specializing in neuropsychology.

#### Feasibility

To assess feasibility, the number of participants enrolled, number of sessions attended and missed, dropout rates and reasons were recorded.

#### Psychopathology

The clinical assessment aimed to verify inclusion and exclusion criteria, including clinical stability at the time of inclusion. The Brief Psychiatric Rating Scale (BPRS), used in schizophrenia and autism (39, 40) to determine psychopathological symptoms and functioning was conducted for each of the participants before and after the program.

#### Neurocognition

This assessment included several standardized tests: the D2-R Attention Test, which measures sustained attention and processing speed (41); the Trail Making Test, assessing visual attention, cognitive flexibility, and executive functioning (42); the Digit Span subtest of the WAIS-IV, evaluating working memory (43); and the Stroop Color–Word Test (44), which measures the ability to inhibit cognitive interference by engaging attentional and executive processes. Verbal episodic memory was evaluated using the California Verbal Learning Test (CVLT; 45). Planification was assessed using the Commissions Revised Test (46).

#### Social cognition

To evaluate baseline and post-program performance, social cognition was assessed using the ClaCoS battery (47). The ClaCoS was validated by a French network of neuropsychologists and psychiatrists, specialized in psychosocial rehabilitation and research groups. It has been validated in schizophrenia, autism, bipolar disorders and alcoholic dependence (36, 48–51). The battery includes: a self-report questionnaire assessing social cognition complaints (ACSo, Self-Assessment of Social Cognition; 50); a facial emotion recognition test (TREF; 52); a questionnaire evaluating the analysis of social contexts and conventions (PERSO, Social Perception and Knowledge; 47); a theory-of-mind test (MASC, Movie for the Assessment of Social Cognition; 35, French adaptation by 36); and a questionnaire measuring attributional biases (AIHQ, Ambiguous Intentions and Hostility Questionnaire; 53).

The ACSo is a 12-item self-assessment questionnaire designed to assess different social cognition difficulties in encountered in daily life. Each item is rated on a Likert scale ranging from 0 (never) to 4 (very often). The questionnaire yields a total score reflecting overall social cognition complaints, along with four subscale scores assessing difficulties in emotion perception, social perception and knowledge, theory of mind, and attributional style.

The TREF is a facial emotion identification test, with a presentation of 54 faces representing 6 different emotions: joy, fear, sadness, anger, disgust and contempt. Each emotion is shown at intensity levels ranging from 20% to 100% using morphing. A global identification score is computed, as well as emotion-specific accuracy scores and average detection thresholds for each emotion.

The PERSO assesses social perception, contextual analysis, and social knowledge. Participants are presented with four illustrated boards depicting social situations. They are first asked to freely describe and analyze the scene, then to identify any elements they may have missed, and finally to specify the social convention associated with the situation (e.g., giving up one’s seat to an older adult on public transportation).

The MASC is a 45-minute film-based measure of theory-of-mind abilities. During the film, 45 multiple-choice questions are presented, each probing the characters’ feelings, thoughts, or intentions at the moment of questioning. For each item, the four response options correspond to an accurate mental-state inference (ToM), an over-interpretation (Hyper-ToM), an under-interpretation (Hypo-ToM), or an answer that does not consider mental states (No-ToM).

The AIHQ includes five ambiguous scenarios in which participants are asked to imagine themselves and respond to five questions assessing hostility bias, responsibility attribution, and aggressiveness. Hostility and aggressiveness biases are evaluated through two open-ended questions, while responsibility attribution is assessed via three items rated on 5- or 6-point Likert scales.

#### Global functioning

Global functioning was assessed using the Global Assessment of Functioning (DMS-4, APA, 1994).

The GAF is a clinical scale for measuring psychological, social and occupational functioning on a scale of 0 to 100, subdivided into 10-point increments. Higher scores indicate the absence of symptoms of a physical or mental disorder and satisfactory functioning, while lower scores reflect substantial symptomatology with marked impairment in daily functioning and autonomy. The score is determined by the trained evaluator based on clinical elements observed and reported during the patient interview.

#### Social functioning

Difficulties in social functioning and social interactions were assessed using the Functional Repercussion Scale – Social Cognition (ERF-CS; 54) and the Self-Assessment of Conversational Skills (AHC; 55). The ERF-CS is a 14-item semi-structured interview that evaluates the functional impact in daily life of difficulties across four social cognition domains: emotion perception, social perception and knowledge, theory of mind, and attributional biases. Examples of everyday difficulties are provided, and participants indicate whether they experience each one. If so, they are asked to elaborate and provide examples of similar situations. They then rate both the frequency of the difficulty (1 = exceptionally, 5 = almost daily) and the level of associated distress (1 = not at all distressing, 5 = extremely distressing) using two Likert scales.

The AHC is a self-report scale assessing perceived difficulties in social interaction. It consists of 16 items corresponding to statements such as “I know when I can say intimate things and when I can’t”. Participants rate the extent to which each statement applies to them on a 4-point Lickert scale (1=no, 4=absolutely).

### Statistical analysis

Descriptive statistics are presented as means and standard deviations for continuous variables, and as counts and percentages for categorical variables. Concerning clinical and neuropsychological performance before and after PICSIS, the prospective design of this study only allows comparison of participants’ performance before and after the program. Changes in scores between pre- and post-program assessments were evaluated using the Wilcoxon signed-rank test, a non-parametric test suitable for our data sample, given the small number of participants and the data’s heterogeneity. Alongside, the median difference was estimated using the Hodges-Lehmann estimator, coupled with a 95% confidence interval to provide a robust and interpretable measure of central tendency and precision. Effect size was calculated as the rank-biserial correlation r. The sign of r indicates the direction of change and the absolute value represents the magnitude of the effect(small: 0.1-0.3; moderate: 0.3-0.5; large: >0.5).

## Results

### Sociodemographic, clinical, and cognitive characteristics of participants

The program was administered to 22 participants distributed across four groups. Two participants discontinued prematurely, both due to lack of availability: one after one session and the other one after completing the emotion module. In addition, one participant completed the sessions but declined to have his data used for research purpose, and another one was hospitalized for a manic episode during the program and was therefore excluded for the analysis. The final sample consisted of 18 participants, including 4 women.

Among the 18 participants, eight had a psychotic disorder (schizophrenia or schizoaffective disorder), ten were diagnosed with ASD, and two of these had comorbid ASD and psychosis. Three participants with autism also presented with attention deficit hyperactivity disorder (ADHD), though none received ADHD treatment due to adequate neurocognitive performance. Sociodemographic, clinical, therapeutic, functional, and social cognition characteristics are presented in **Table 1**. Baseline neurocognitive descriptive data, not central to the objectives of this paper, are provided in **Supplementary Material S1**.

**Table 1:** Sociodemographic, diagnostic, treatments data and neurocognitive performance.

Variables	Group (n=18) Mean (SD)
Age	35.83 (9.30)
Education (years)	14.05 (2.46)
Gender (% male)	14/18 (77%)
Diagnosis ([n] %)	
- Schizophrenia	[8] 44%
- ASD	[4] 22%
- ASD + Schizophrenia	[2] 11%
- ASD + ADHD	[3] 17%
Treatment ([n] %)	
- Antidepressant	[3] 17%
- Antipsychotic	[4] 22%
- Antipsychotic + Antidepressant	[3] 17%
- Antipsychotic + Mood stabilizer	[2] 11%
- Antipsychotic + Antidepressant + Mood stabilizer	[2] 11%
- Antipsychotic + other treatment	[1] 5%
- Anxiolytic	[0]
- No treatment	[3] 17%
BPRS scores	39.28 (12.60)

ASD, Autism Spectrum Disorder; ADHD, Attention Deficit/Hyperactivity Disorder.

Of the 18 participants, four with a schizophrenia diagnosis had completed the NEAR program (Neuropsychological Educational Approach to Remediation), a group-based neurocognitive remediation method (56), within the previous 18 months; none of the participants with autism had completed this method. Importantly, NEAR does not include social cognition training.

#### Missing data

The neuropsychological evaluation of social cognition was conducted across two successive assessment sessions. In some cases, participants failed to attend one of the sessions, resulting in missing data. Overall, 16 out of 216 (7,41%) data points were missing in ACSO, PERSO and ERF-CS, with missingness ranging from 5.5% for ACSO to 22.22% for ERF-CS scores.

#### Tolerability

The mean attendance rate across the 30 sessions was 89.44%, with individual rates ranging from 66.7% to 100%. Absences were notified at least on the same day and were due to viral infections, transportation problems, academic exams or vacations.

#### Preliminary results

Results are presented in **Table 2**. Clinical symptoms did not significantly changed between T0 and T1 [BPRS: T0 = 39.28 (12.60); T1 = 36.61 (9.80) p=0.312].

**Table 2:** Differences in psychopathology, global functioning and social cognition before and after the PICSIS intervention.

Measure	T0		T1		p-value	Rank biserial correlation(Effect size) - r	Confidence interval	Hodges-Lehmann Estimator
	moy	s.d.	moy	s.d.				
BPRS	39.28	12.60	36.61	9.80	0.312	-0.242	[-7.5, 2.5]	-2.5
GAF	62.06	14.136	67.00	13.05	0.443	0.433	[-5, 12.5]	2.5
ERF Social cognition	43.77	26.89	34.62	29.11	0.083	-0.606	[-22, 1]	-12.41
AHC	24.50	12.55	26.89	9.79	0.185	0.373	[-2, 7]	3.00
ACSO	18.75	8.55	14.75	6.74	0.0037*	-0.886	**[-7, -2]**	**-4.5**
TREF	60.47	15.25	68.04	6.62	0.070	0.522	[-0.45, 13]	5.56
PERSO perception score	18.63	1.821	18.81	2.90	0.752	0.100	[-1.5, 2]	0.91
PERSO social knowledge	3.88	1.89	5.13	1.99	0.092	0.500	[0.005, -3]	1.5
MASC	23.59	3.79	26.24	5.82	0.033*	0.610	**[0.001, 5]**	**2.5**
AIHQ hostility bias	1.89	0.72	1.52	0.61	0.009*	-0.824	**[-0.8, -0.1]**	**-0.40**
AIHQ responsibility bias	2.65	0.91	2.48	0.91	0.331	-0.275	[-0.55, 0.25]	-0.15
AIHQ aggressivity bias	1.71	0.63	1.43	0.41	0.086	-0.508	[-0.6, 0.1]	-0.33

Wilcoxon signed rank with continuity correction, Hodges-Lehmann estimation. GAF, Global Assessment Scale; ERF, Echelle de Répercussions Fonctionnelle; AHC, Autoévaluation des Habiletés Conversationnelles; ACSO, Autoévaluation de la Cognition Sociale; TREF, Test de Reconnaissance des Emotions Faciales; PERSO, Test d’évaluation de la PERception et des connaissances SOciale; MASC, Movie for the Assesment of Social Cognition; AIHQ, the Ambiguous Intentions Hostility Questionnaire. Bold values are visual mark associated with a significant p-value.

Similarly, no significant difference was observed in global functioning [GAF: T0 = 62.06 (14.136); T1 = 67.00 (13.05) p=0.443].

There was a significant reduction in overall social cognition complaints [ACSO: T0 = 18.75 (8.55); T1 = 14.75 (6.74); p=0.0037, r = -0.886]. The Hodges-Lehmann estimator indicated a median difference of -4.5 points between pre- and post-program scores (95% CI [-7; -2]), demonstrating a meaningful decrease following the intervention.

No significant improvement was observed in facial emotion recognition [TREF: T0 = 60.47 (15.25); T1 = 68.04 (6.62); p=0.070, r = -0.522], nor in the analysis of social contexts or social knowledge on the PERSO.

Theory of mind performance, measured by the MASC, showed a significant improvement [MASC: T0 = 23.59 (3.79); T1 = 26.24 (5.82); p=0.033, r = -0.610]. The Hodges-Lehmann estimator indicated a median increase of +2.5 points (95% CI [0.001; 5]).

For the AIHQ, the Hodges-Lehmann estimator indicated a median reduction of -0.45 points in hostility attribution (95% CI [-0.8; -0.1]), reflecting a significant decrease [Hostility Bias: T0 = 1.89 (0.72); T1 = 1.52 (0.61); p=0.009, r = -0.824]. No significant change was observed neither for aggressivity bias [Aggressivity Bias: T0 = 1.71 (0.63); T1 = 1.43 (0.41); p=0.086, r = -0.508] nor for responsibility attribution bias [Responsibility Bias: T0 = 2.65 (0.91); T1 = 2.48 (0.91); p=0.331, r = -0.275].

Regarding functional difficulties, no significant change was found in total social cognition impact [ERF-CS: T0 = 43.77 (26.89); T1 = 34.62 (29.11); p=0.083, r = -0.606].

## Discussion

The aim of this preliminary study was to assess the feasibility and initial effects of an innovative program combining SCR and SST. Given the level of engagement required over the 30 sessions, the low rate of non-attendance provides an indirect indicator of feasibility.

No significant effect was observed on clinical symptoms as measured by the BPRS. This absence of clinical change was expected, as cognitive remediation programs—whether targeting neurocognition or social cognition—primarily aim to improve cognitive skills rather than reduce symptom severity, and are typically delivered to clinically stable participants. Baseline mean scores were consistent with previous studies involving clinically stable individuals enrolled in rehabilitation programs (57).

Improvements emerged in theory of mind performance and in cognitive biases, particularly hostility attribution. Reductions in self-reported social cognition complaints also indicated better perceived social-cognitive functioning, although these changes did not extend to functional outcomes. These results align with the strategies practiced throughout the PICSIS sessions. However, despite improvements in perceived difficulties, no significant gains were observed in social scene analysis or in overall functioning and conversational skills. The preliminary benefits identified were modest but consistent with effects reported in similar integrative programs (10), with no specific improvements in social analysis.

Unlike other broad-based interventions such as CET (21, 23) or ISST (26), PICSIS targets all four core components of social cognition (FEI, SP/SK, ToM, and AB) in combination with SST. Its objective is not to address disorder-specific difficulties, as programs like SCIT-S (13) or SCIT-A (58) do, but rather to offer a functional, transdiagnostic intervention grounded in the social cognition and SST literature. For this study, PICSIS was administered to individuals with schizophrenia and to individuals with ASD without intellectual disability. Results suggest good acceptability and feasibility, as well as changes in several social-cognitive processes.

The alternation of remediation and social skills sessions created a dynamic structure suited to everyday functioning and appeared to enhance the integration of theoretical concepts. Participants expressed high satisfaction with the program, as reflected in attendance rates. The use of real-life situations reported by the participants themselves also facilitated understanding and transfer to daily life, and was frequently described as highly motivating. The group composition—bringing together individuals with schizophrenia and ASD within a transdiagnostic framework (59)—supported rich interactions, mutual support, and the sharing of complementary perspectives. If confirmed in larger samples, such a transdiagnostic approach could contribute to destigmatization (60) and improve the lived experience of mental disability and social difficulties (61).

PICSIS positions itself as an integrative tool designed to address both social cognition and social interaction difficulties. With 30 two-hour sessions for groups of 4–8 participants, it provides a comprehensive program targeting all domains of social cognition and offering social skills practice, particularly useful for individuals with broad or multiple social cognitive impairments. Its design, which allows adaptation to diverse cognitive profiles, may reduce human and material costs in specialized care settings.

This pilot study has several limitations. First, it will be necessary to test the program in a larger sample and within a randomized controlled design to demonstrate its efficacy. Second, it may be relevant to analyze outcomes according to social-cognitive profiles—such as ToM deficits vs. ToM excess as assessed by the MASC—independently of diagnosis. Third, as an open-label ecological study with a small sample, variability is considerable; furthermore, the mixed diagnostic composition limits the ability to attribute effects to specific populations. It would therefore be valuable to examine PICSIS separately in larger samples of individuals with schizophrenia and ASD. Fourth, participation in a neurocognitive remediation program within the previous 18 months may have influenced social cognition or functional outcomes. Finally, treatment status in schizophrenia and ASD, as well as comorbidities among participants with ASD, may introduce confounding variables contributing to population heterogeneity.

## Conclusion

In conclusion, this very preliminary study suggests that the PICSIS program is a broad-based, integrative intervention capable of addressing a wide range of social difficulties, from social cognition to social interaction. When applied to young adults, PICSIS may help prevent the progression of social cognition impairments in schizophrenia—impairments that can substantially affect long-term outcomes and may support improvements in the core social difficulties observed in autism, thereby fostering social and vocational inclusion.

Future research should involve a larger sample within a randomized controlled design, including the examination of potential confounding variables, to confirm the program’s efficacy and to better delineate its role within the spectrum of psychosocial interventions. PICSIS has the potential to provide comprehensive support for cognitive and social interaction difficulties through group-based work, offering both clinical and organizational advantages and broad applicability for individuals with mental disabilities and disabling social cognitive deficits.

Our findings raise the question of PICSIS’s utility for both over-interpretive social-cognitive profiles, commonly observed in schizophrenia, and under-interpretive profiles, typically found in ASD, without requiring adaptation of the program content. A large-scale validation study is needed to confirm acceptability and effectiveness across these two distinct cognitive profiles. Such confirmation would further support the potential contribution of PICSIS to health-economic efficiency.

## Data Availability

The raw data supporting the conclusions of this article will be made available by the authors, without undue reservation.

## References

[1] World Health Organization . (2025). Autism. Available online at: https://www.who.int/news-room/fact-sheets/detail/autism-spectrum-disorders.

[2] Bagheri S Yu JC Gallucci J Tan V Oliver LD Dickie EW . Transdiagnostic neurobiology of social cognition and individual variability as measured by fractional amplitude of low-frequency fluctuation in autism and schizophrenia spectrum disorders. Biol Psychiatry: Cogn Neurosci Neuroimaging. (2025). doi: 10.1016/j.bpsc.2025.04.004, PMID: 40268245 PMC12353298

[3] Ozbek SU Sut E Bora E . Comparison of social cognition and neurocognition in schizophrenia and autism spectrum disorder: A systematic review and meta-analysis. Neurosci Biobehav Rev. (2023) 155:105441. doi: 10.1016/j.neubiorev.2023.105441, PMID: 37923237

[4] Gur RC Gur RE . Social cognition as an RDoC domain. Am J Med Genet Part B: Neuropsychiatr Genet. (2016) 171:132–41. doi: 10.1002/ajmg.b.32394, PMID: 26607670 PMC4843508

[5] Barlati S Minelli A Ceraso A Nibbio G Carvalho Silva R Deste G . Social cognition in a research domain criteria perspective: a bridge between schizophrenia and autism spectra disorders. Front Psychiatry. (2020) 11:806. doi: 10.3389/fpsyt.2020.00806, PMID: 33005149 PMC7485015

[6] Halverson TF Orleans-Pobee M Merritt C Sheeran P Fett A-K Penn DL . Pathways to functional outcomes in schizophrenia spectrum disorders: Meta-analysis of social cognitive and neurocognitive predictors. Neurosci Biobehav Rev. (2019) 105:212−219. doi: 10.1016/j.neubiorev.2019.07.020, PMID: 31415864

[7] Mucci A Galderisi S Gibertoni D Rossi A Rocca P Bertolino A . Factors associated with real-life functioning in persons with schizophrenia in a 4-year follow-up study of the Italian network for research on psychoses. JAMA Psychiatry. (2021) 78:550–9. doi: 10.1001/jamapsychiatry.2020.4614, PMID: 33566071 PMC7876615

[8] Galderisi S Rossi A Rocca P Bertolino A Mucci A Bucci P . The influence of illness-related variables, personal resources and context-related factors on real-life functioning of people with schizophrenia. World Psychiatry. (2014) 13:275–87. doi: 10.1002/wps.20167, PMID: 25273301 PMC4219069

[9] Wong TR Boulton KA Demetriou EA Thomas EE Phillips NL Hankin L . Executive Function and Social Cognition Performance Predicts Social Difficulty for Autistic Adults. Autism Research. (2025) 18:1734–45. doi: 10.1002/aur.70090, PMID: 40747689 PMC12442527

[10] Roelofs RL Wingbermühle E Egger JIM Kessels RPC . Social cognitive interventions in neuropsychiatric patients: A meta-analysis. Brain Impairment. (2017) 18:138−173. doi: 10.1017/BrImp.2016.31

[11] Bazin N Passerieux C Hardy-Bayle M-C . ToMRemed : Une technique de remédiation cognitive centrée sur la théorie de l’esprit pour les patients schizophrènes. J Thérapie Comportementale Cogn. (2010) 20:16−21. doi: 10.1016/j.jtcc.2010.02.001

[12] Gaudelus B Franck N . Troubles du traitement des informations faciales. Le programme Gaïa. In: La remédiation cognitive. France: Elsevier Masson (2012). p. 169−181.

[13] Combs DR Adams SD Penn DL Roberts D Tiegreen J Stem P . Social Cognition and Interaction Training (SCIT) for inpatients with schizophrenia spectrum disorders: Preliminary findings. Schizophr Res. (2007) 91:112−116. doi: 10.1016/j.schres.2006.12.010, PMID: 17293083

[14] Fiszdon JM Dixon HD Davidson CA Roberts DL Penn DL Bell MD . Efficacy of social cognition and interaction training in outpatients with schizophrenia spectrum disorders: Randomized controlled trial. Front Psychiatry. (2023) 14:1217735. doi: 10.3389/fpsyt.2023.1217735, PMID: 37599886 PMC10436290

[15] Peyroux É. Franck N . RC2S: A cognitive remediation program to improve social cognition in schizophrenia and related disorders. Front Hum Neurosci. (2014) 8:400. doi: 10.3389/fnhum.2014.00400, PMID: 24982627 PMC4055942

[16] Roberts DL Penn DL . Social cognition and interaction training (SCIT) for outpatients with schizophrenia: A preliminary study. Psychiatry Res. (2009) 166:141−147. doi: 10.1016/j.psychres.2008.02.007, PMID: 19272654

[17] Peyroux E Franck N . Improving Social Cognition in People with Schizophrenia with RC2S: Two Single-Case Studies. Front Psychiatry. (2016) 7:66. doi: 10.3389/fpsyt.2016.00066, PMID: 27199776 PMC4842761

[18] Peyroux E Franck N . Is social cognitive training efficient in autism? A pilot single-case study using the RC2S+ program. Neurocase. (2019) 25:217−224. doi: 10.1080/13554794.2019.1666877, PMID: 31522609

[19] Bowie C Mcgurk S Mausbach B Patterson T Harvey P . Combined cognitive remediation and functional skills training for schizophrenia: effects on cognition, functional competence, and real-world behavior. Am J Psychiatry. (2012) 169:710–8. doi: 10.1176/appi.ajp.2012.11091337, PMID: 22581070

[20] Hogarty GE Flesher S . Practice principles of cognitive enhancement therapy for schizophrenia. Schizophr Bull. (1999) 25:693−708. doi: 10.1093/oxfordjournals.schbul.a033411, PMID: 10667740

[21] Eack SM Hogarty SS Greenwald DP Litschge MY Porton SA Mazefsky CA . Cognitive enhancement therapy for adult autism spectrum disorder: Results of an 18-month randomized clinical trial. Autism Res. (2018) 11:519−530. doi: 10.1002/aur.1913, PMID: 29286586 PMC5867220

[22] Hogarty GE Flesher S Ulrich R Carter M Greenwald D Pogue-Geile M . Cognitive Enhancement Therapy for Schizophrenia: Effects of a 2-Year Randomized Trial on Cognition and Behavior. Arch Gen Psychiatry. (2004) 61:866–76. doi: 10.1001/archpsyc.61.9.866, PMID: 15351765

[23] Eack SM Greenwald DP Hogarty SS Cooley SJ DiBarry AL Montrose DM . Cognitive enhancement therapy for early-course schizophrenia: Effects of a two-year randomized controlled trial. Psychiatr Serv (Washington D.C.). (2009) 60:1468−1476. doi: 10.1176/appi.ps.60.11.1468, PMID: 19880464 PMC3693549

[24] Turner DT McGlanaghy E Cuijpers P van der Gaag M Karyotaki E MacBeth A . A meta-analysis of social skills training and related interventions for psychosis. Schizophr Bull. (2018) 44:475−491. doi: 10.1093/schbul/sbx146, PMID: 29140460 PMC5890475

[25] Hotton M Coles S . The effectiveness of social skills training groups for individuals with autism spectrum disorder. Rev J Autism Dev Disord. (2016) 3:68−81. doi: 10.1007/s40489-015-0066-5

[26] Wölwer W Frommann N Lowe A Kamp D Weide K Bechdolf A . Efficacy of integrated social cognitive remediation vs. Neurocognitive remediation in improving functional outcome in schizophrenia: concept and design of a multicenter, single-blind RCT (The ISST study). Front Psychiatry. (2022) 13:909370. doi: 10.3389/fpsyt.2022.909370, PMID: 35800017 PMC9253387

[27] Kamp D Lowe A Weide K Riesbeck M Bechdolf A Leopold K . Efficacy of integrated social cognitive remediation vs neurocognitive remediation in schizophrenia: Results from the multicenter randomized controlled ISST (Integrated Social Cognition And Social Skills Therapy) study. Schizophr Res. (2025) 277:44−56. doi: 10.1016/j.schres.2025.02.015, PMID: 40015077

[28] Barlati S Nibbio G Vita A . Evidence-based psychosocial interventions in schizophrenia: A critical review. Curr Opin Psychiatry. (2024) 37:131–9. doi: 10.1097/YCO.0000000000000925, PMID: 38410981 PMC10990032

[29] Gantman A Kapp SK Orenski K Laugeson EA . Social skills training for young adults with high—functioning autism spectrum disorders: A randomized controlled pilot study. J Autism Dev Disord. (2012) 42:1094–103. doi: 10.1007/s10803-011-1350-6, PMID: 21915740

[30] McVey AJ Dolan BK Willar KS Pleiss S Karst JS Casnar CL . A replication and extension of the PEERS for young adults social skills intervention: Examining effects on social skills and social anxiety in young adults with autism spectrum disorder. J Autism Dev Disord. (2016) 46:3739–54. doi: 10.1007/s10803-016-2911-5, PMID: 27628940 PMC5310211

[31] Gorenstein M Giserman-Kiss I Feldman E Isenstein EL Donnelly L Wang AT . Breif report: A Job-Based Social Skills Program (JOBSS) for adults with Autism Spectrum Disorder: A pilot randomized controlled trial. J Autism Dev Disord. (2020) 50:4527–34. doi: 10.1007/s10803-020-04482-8, PMID: 32297122

[32] Morgan L Leatzow A Clark S Siller M . Interview skills for adults with autism spectrum disorder: A pilot randomized controlled trial. J Autism Dev Disord. (2014) 44:2290–300. doi: 10.1007/s10803-014-2100-3, PMID: 24682707

[33] Yeo H Yoon S Lee J Kurtz MM Choi K . A meta-analysis of the effects of social-cognitive training in schizophrenia: The role of treatment characteristics and study quality. Br J Clin Psychol. (2022) 61:37−57. doi: 10.1111/bjc.12320, PMID: 34291465

[34] Le Couteur A Haden G Hammal D McConachie H . Diagnosing Autism Spectrum Disorders in pre-school children using two standardised assessment instruments: The ADI6R and the ADOS. J Autism Dev Disord. (2008) 38:362–72. doi: 10.1007/s10803-007-0403-3, PMID: 17605097

[35] Dziobek I Fleck S Kalbe E Rogers K Hassenstab J Brand M . Introducing MASC: A movie for the assessment of social cognition. J Autism Dev Disord. (2006) 36:623−636. doi: 10.1007/s10803-006-0107-0, PMID: 16755332

[36] Martinez G Alexandre C Mam-Lam-Fook C Bendjemaa N Gaillard R Garel P . Phenotypic continuum between autism and schizophrenia: Evidence from the Movie for the Assessment of Social Cognition (MASC). Schizophr Res. (2017) 185:161−166. doi: 10.1016/j.schres.2017.01.012, PMID: 28089135

[37] Medalia AA Freilich B . The Neuropsychological Educational Approach to Cognitive Remediation (NEAR) Model: Practice Principles and Outcome Studies. Am J Psychiatr Rehabil. (2008) 11:123–43. doi: 10.1080/15487760801963660

[38] Bowie CR Bell MD Fiszdon JM Johannesen JK Lindenmayer J-P McGurk SR . Cognitive remediation for schizophrenia: An expert working group white paper on core techniques. Schizophr Res. (2020) 215:49−53. doi: 10.1016/j.schres.2019.10.047, PMID: 31699627

[39] The Brief Psychiatric Rating Scale (BPRS) . Recent developments in ascertainment and scaling. Psychopharmacology Bulletin. (1988) 24:97–100. 3387516

[40] Rausch JL Sirota EL Londino DL Johnson ME Carr BM Bhatia R . Open-label risperidone for Asperger’s disorder: Negative symptom spectrum response. J Clin Psychiatry. (2005) 66:1592–7. doi: 10.4088/JCP.v66n1216, PMID: 16401163

[41] Zillmer RBE Brickenkamp R . The d2 test of attention. France: Hogrefe and Huber Publishers (1998).

[42] Godefroy O GREFEX O . Executive functions and neurological and psychiatric pathologies: Evaluation in clinical practice. Marseille: Éditions Solal (2008).

[43] Wechsler D . Wechsler adult intelligence scale WAIS-IV Canadian. Canada: Pearson (2008).

[44] Stroop JR . Studies of interference in serial verbal reactions. J Exp Psychol. (1935) 18:643. doi: 10.1037/h0054651

[45] Woods SP Delis DC Scott JC Kramer JH Holdnack JA . The California Verbal Learning Test–second edition: Test-retest reliability, practice effects, and reliable change indices for the standard and alternate forms. Arch Clin Neuropsychol. (2006) 21:413–20. doi: 10.1016/j.acn.2006.06.002, PMID: 16843636

[46] Fournet N Demazières-Pelletier Y Favier S Lemoine L Gros C . Test des commissions révisé. In: Dans Hugonot-Diener L Thomas-Antérion C Sellal F , editors. GREMOIRE 2 tests et échelles des maladies neurologiques avec symptomatologie cognitive. France: De boeck solal (2015). p. 70–4.

[47] Collectif Clacos . ClaCoS : Consensus autour de la Cognition Sociale, Manuel. France: Hogrefe (2022).

[48] Morel-Kohlmeyer S Thillay A Roux S Amado I Brenugat L Carteau-Martin I . When alterations in social cognition meet subjective complaints in autism spectrum disorder: evaluation with the « ClaCoS » Battery. Front Psychiatry. (2021) 12:643551. doi: 10.3389/fpsyt.2021.643551, PMID: 34512407 PMC8426662

[49] Peyroux E Prost Z Danset-Alexandre C Brenugat-Herne L Carteau-Martin I Gaudelus B . From “under” to “over” social cognition in schizophrenia: Is there distinct profiles of impairments according to negative and positive symptoms? Schizophrenia Research: Cognition. (2018) 15:21–9. doi: 10.1016/j.scog.2018.10.001, PMID: 30534527 PMC6260279

[50] Graux J Thillay A Morlec V Sarron P-Y Roux S Gaudelus B . A transnosographic self-assessment of social cognitive impairments (ACSO): first data. Front Psychiatry. (2019) 10:847. doi: 10.3389/fpsyt.2019.00847, PMID: 31824350 PMC6881457

[51] Maurage P Pabst A Lannoy S d’Hondt F De Timary P Gaudelus B . Tackling heterogeneity: Individual variability of emotion decoding deficits in severe alcohol use disorder. J Affect Disord. (2021) 279:299–307. doi: 10.1016/j.jad.2020.10.022, PMID: 33096328 PMC7738413

[52] Gaudelus B Virgile J Peyroux E Leleu A Baudouin J-Y Franck N . Measuring impairment of facial affects recognition in schizophrenia. Preliminary study of the facial emotions recognition task (TREF). L’Encephale. (2015) 41:251−259. doi: 10.1016/j.encep.2014.08.013, PMID: 25240938

[53] Combs DR Penn DL Wicher M Waldheter E . The Ambiguous Intentions Hostility Questionnaire (AIHQ): A new measure for evaluating hostile social-cognitive biases in paranoia. Cogn Neuropsychiatry. (2007) 12:128−143. doi: 10.1080/13546800600787854, PMID: 17453895

[54] Gaudelus B Peyroux E Colson S Franck N . L’évaluation des répercussions fonctionnelles des altérations de la cognition sociale favorise-t-elle l’engagement dans les soins des personnes ayant des troubles psychotiques? Annales Médico-psychologiques Rev psychiatrique. (2018) 176:94−99. doi: 10.1016/j.amp.2017.11.003

[55] Pomini V . Echelle d’autoévaluation des habiletés conversationnelles (AHC) [questionnaire]. (1999). Available online at: https://www.calameo.com/sante-mentale-psychoeducation/read/00726237063bc66a15344.

[56] Medalia A Herlands T Saperstein AM Revheim N . Cognitive remediation of psychological disorders: Therapist guide. 2nd éd. United States: Oxford University Press (2017).

[57] Harvey PD Strassnig M . Predicting the severity of everyday functional disability in people with schizophrenia: Cognitive deficits, functional capacity, symptoms, and health status. World Psychiatry. (2012) 11:73–9. doi: 10.1016/j.wpsyc.2012.05.004, PMID: 22654932 PMC3363376

[58] Turner-Brown LM Perry TD Dichter GS Bodfish JW Penn DL . Brief report: feasibility of social cognition and interaction training for adults with high functioning autism. J Autism Dev Disord. (2008) 38:1777−1784. doi: 10.1007/s10803-008-0545-y, PMID: 18246419 PMC2646378

[59] Amado I Sederer LI . Implementing cognitive remediation programs in France: the “secret sauce. Psychiatr Serv. (2016) 67:707–9. doi: 10.1176/appi.ps.201600033, PMID: 26975526

[60] Richa S Choueifati D Chemali N Amado I . Les enjeux éthiques de la réhabilitation psychosociale. L’Encéphale. (2024) 50:348–50. doi: 10.1016/j.encep.2023.09.003, PMID: 38423859

[61] Amado I Moualla M Jouve J Brénugat-Herné L Attali D Willard D . Employment, studies and feelings: two to nine years after a personalized program of cognitive remediation in psychiatric patients. Front Psychiatry. (2020) 11:609. doi: 10.3389/fpsyt.2020.00609, PMID: 32733290 PMC7358613

