# Supplementary material for: Integrative social cognition remediation with social skills training for adults: PICSIS – pilot study in autism and schizophrenia
Source: Front Psychiatry. 2026 Jan 12;16:1688937. doi: 10.3389/fpsyt.2025.1688937 (PMC12832802; doi:10.3389/fpsyt.2025.1688937)
Supplement: Supplementary file 1 [file Table1.docx]

S 1 : Baseline Neurocognitive descriptive data

| **Neurocognitive tests** | **Mean** | **Standard Deviations** |
| --- | --- | --- |
| **D2** PS | 280.25 | 136.01 |
| **D2** Perf | 6.00 | 4.52 |
| **D2** Conc | 145.56 | 30.51 |
| **TMT** A | 36.31 | 7.90 |
| **TMT** B | 83.25 | 34.53 |
| **TMT** B-A | 43.19 | 30.20 |
| **Digit Span** Std Scores | 10.18 | 2.92 |
| **Stroop** R T | 46 | 14.93 |
| **Stroop** DT | 68.19 | 19.68 |
| **Stroop** IT | 107.06 | 26.60 |
| **Stroop** D-I | 38.88 | 17.31 |
| **CVLT** R1 | 6.76 | 2.25 |
| **CVLT** R1-5 | 54.94 | 9.68 |
| **CVLT** STFR | 11.53 | 3.32 |
| **CVLT** LTFR | 12.12 | 3.18 |
| **CRT** CT | 9.86 | 3.24 |
| **CRT** TRB | 2.29 | 1.57 |

**Note.** **D2 : D2 test** ; D2 PS : D2 Processing Speed ; D2 Perf : D2 Performance Score ; D2 Conc : D2 Concentration score ; **TMT : Trail Making Test** ; TMT-A : TMT form A ; TMT-B : TMT Form B ; TMT B-A : Substraction Time formB-formA ; **Digit Span : Digit Span test of the WAIS IV test**; Digit Span Sd Score : Digit Span Standard Score ; **Stroop : Stroop Color Naming Test** ; Stroop RT : Stroop Reading Time ; Stroop DT : Stroop Denomination time ; Stroop IT: Stroop Interference Time; Stroop D-I : Stroop Denomination – Interference ; **CVLT : California Verbal Naming Test** ; CVLT R1 : CVLT first Recall ; CVLT R1-5 : CVLT first to 5th Recall ; CVLT STFR : CVLT Short Term Free Recall ; CVLT LTFR : CVLT Long Term Free Recall ; **CRT:** Commissions Revised Test ; CRT CT : MET Completion Time ; MET TotRB : CRT Total Rule Breaks.
